# Impact of inhalation therapy on the incidence of carious lesions in patients with asthma and COPD

**DOI:** 10.1590/1678-7757-2016-0147

**Published:** 2017

**Authors:** Branislava Velicki Bozejac, Ivana Stojšin, Mirna Ðuric, Biljana Zvezdin, Tatjana Brkanić, Evica Budišin, Karolina Vukoje, Nevena Sečen

**Affiliations:** 1 Stomatološka poliklinika Dentaland Novi Sad, Srbija; 2 Medicinski fakultet Novi Sad, Univerzitet u Novom Sadu, Srbija; 3 Odeljenje za bolesti zuba i endodonciju, Klinika za stomatologiju Vojvodine, Novi Sad, Srbija; 4 lnstitut za plućne bolesti Vojvodine, Sremska Kamenica, Srbija

**Keywords:** Dental caries, Asthma, Chronic obstructive pulmonary diseases, Saliva, Salivation, Inhalation therapy

## Abstract

**Objective:**

The aim of this study was to investigate the incidence of carious lesions, the amount of salivary flow rate and pH value in patients with asthma and chronic obstructive pulmonary diseases (COPD), using inhalation therapy. The obtained results were compared with the results of adult healthy subjects, forming a control group.

**Material and Methods:**

The study included 80 participants aging between 18 and 65 years. The experimental group (EG) was comprised of 40 participants, previously diagnosed with asthma or COPD undergoing inhalation therapy for more than five years. The control group (CG), comprised of 40 participants, mirrored the same age and gender status of the EG. Dental status was determined by decayed, missing, and filled teeth (DMFT index). Quantity and pH value of saliva were determined in the laboratory.

**Results:**

In the EG, the mean value of the salivary flow rate and pH value were statistically significantly lower than in the CG (p<0.001). Patients in the EG had a higher value of DMFT index when compared with the CG, although the difference was not statistically significant (p=0.199). Mean number of decayed teeth, as well as missing teeth, in the EG was statistically significantly higher than in the CG (p<0.001). Mean number of filled teeth in the EG was statistically significantly lower than in the CG (p<0.001).

**Conclusion:**

It was found that patients undergoing inhalation therapy face increasing risk of dental caries due to the lower salivary flow rate and pH value along with the inhalation therapy. They should receive intensive preventive care, including oral hygiene instruction and dietary advice.

## Introduction

Dental caries represents a multifactorial disease and it is one of the most prevalent chronic diseases worldwide. Caries lesion forms through a complex interaction between cariogenic oral flora (a biofilm) and fermentable carbohydrates on the tooth surface. The occurrence of carious lesions is an outcome of the disturbed balance between dietetic-bacterial factors and host factors, and the usage of different drugs may significantly influence this misbalance^11^. Based on studies^3^ ^,^ ^6^ ^,^ ^13^ ^,^ ^15^ ^,^ ^24^, patients undergoing inhalation therapy are subjected to higher risk of dental caries, due to the reduced saliva secretion, decreased pH value, enlarged number of cariogenic bacteria caused by inappropriate sugar consumption, insufficient fluoride exposure, poor oral hygiene, and the usage of inhaling medicaments with dry powder. These changes could be related to basic diseases or to the prescribed therapy.

Asthma and chronic obstructive pulmonary diseases (COPD) are predominantly chronic respiratory diseases, the prevalence of which grows in many countries as well as in Serbia (Europe). According to Global Initiative for Asthma^8^, it is estimated that around 400 million people suffer from asthma. The prevalence of asthma in Serbia is 4-5%, and in 2011, for the first time, it was officially published that asthma prevalence on the territory of Serbia was 6.8%^17^. According to Global Initiative for Obstructive Lung Disease, it is estimate that COPD is a leading cause of morbidity and mortality wordlwide^26^. The COPD prevalence has increased from 7% to 14% in the past nine years^28^. The latest data from the Serbian primary health care centers confirmed that the percentage of patients with a clinical suspicion of COPD was 21.9%^27^. The causes and the pathophysiological characteristics of asthma and COPD are different, yet both chronic diseases share common functional characteristics, i.e., a limited air passage through respiratory airways. Inhalation drugs such as beta-2 agonists, anticholinergic bronchodilators, and inhaled corticosteroids have advantage in asthma and COPD therapy^8^ ^,^ ^26^.

The major proportion of the inhaled drug is retained in the oral cavity and oropharynx and it may interfere in the normal physiology of oral tissues^9^. The usage of the inhaled beta-2 agonist in combination with glucocorticoids in asthmatic patients cause the decreased salivary flow rate^18^ and they can reduce the effect of saliva in the aid of protection against caries^21^. When salivary flow is diminished or absent, and the pH value is lower, there is increased food retention, an acidic environment is encouraged and persists longer^9^. Prolonged use of beta-2 agonists is associated with the increased frequency of caries, which could be explained through the basic effect of beta-2 agonists that are connected to the present beta-2 receptors in the parotid and other salivary glands^4^.

The increased susceptibility to dental caries can also be due to the frequent use of dry powder inhaler containing fermentable carbohydrates. The most common is lactose monohydrate, and although it is one of the least cariogenic sugars, it can still lead to an increased dental caries risk^25^. Risk factor for higher caries experience in patients can also be poor hygiene and lack of information about proper maintenance of oral hygiene and preventive measures to preserve dental health. Many authors assessed the influence of inhalation therapy on the oral health in children and adolescents^6^·^15^·^18^·^21^·^23^ while few authors addressed this issue focusing on the adult population^12^ ^,^ ^13^.

Therefore, the main aim of this study was verifying dental caries and saliva characteristics (salivary flow rate, pH value) of patients who suffer from asthma and COPD with inhalation therapy. In addition, we aimed to assess whether participants were informed about proper maintenance of oral hygiene and preventive measures and whether they adhere to them. No such evaluation has been performed in Serbian patients.

## Material and methods

This cross sectional study was approved by the Ethical Committee of the Dental Clinic of Vojvodina, Faculty of Medicine, University of Novi Sad, Serbia (no. 01-47/18-2013). Written informed consent form was obtained from all patients. The study was conducted from 2014 to 2015, and it has included 80 participants aged between 23 and 65. The experimental group (EG) consisted of 40 participants that suffer from asthma or chronic obstructive pulmonary disease (COPD). The diagnosis of asthma and COPD was prepared based on medical anamnesis, clinical examination, and functional tests at the Institute for Pulmonary Diseases of Vojvodina, Novi Sad, Serbia. The main inclusion criterion was the length of inhalation therapy - more than five years. Asthma and COPD were classified as mild, moderate, and severe according to the status of the disease reported by pulmonologists. The EG participants were all using a dry powder or aerosol inhaler. The control group (CG) consisted of 40 healthy participants of the same age, gender status, and in the same geographic area as in the EG. The CG participants were patients treated at the Department of Dentistry and Endodontics, Dental Clinic of Vojvodina, Novi Sad, Serbia. The participants suffering from any other kind of acute or chronic diseases that could influence saliva secretion were excluded from the study. The obtained results have been compared with results from the control group (healthy people). Standard dental anamnesis and dental status of patients were obtained.

### Questionnaire data

A questionnaire was completed by all participants, with the assistance of the dentist, and data were collected for information about patients’ age, gender, dietary habits (the most consumed beverage), oral hygiene habits, use of fluoride (fluoride rinse), their awareness and knowledge of the proper maintenance of oral hygiene, and the frequency of their checkups at the dentist. Anamneses of EG was additionally supported with questions about the type and severity of the diagnosed asthma and COPD, length of treatment, regularity of therapy application, type of used medications, and their habits - wether the patients rinsed the mouth with water after using the inhaler.

### Clinical caries examination

Dental examination was carried out by three dentists. Three examiners performed data collection, one responsible for the questioner, another for saliva determination (salivary flow rate and salivary pH value), and the last one for the dental diagnosis. The examiners were blinded to the subject's group assignment. Dental caries diagnosis was performed according to WHO criteria^29^, and DMFT index for permanent teeth (number of decayed, missing, and filled teeth) were calculated. Checkup was made under standard dental control using a dental mirror and dental probes with artificial illumination. A clinical study contained a dental checkup comprising participants’ health status of hard dental tissues and the presence of manifest carious lesions. Radiograph was not included in the examination.

### Salivary and pH analyses

Laboratory investigation comprised registering the salivary flow rates of non-stimulated saliva and the pH value. Before the saliva sample completion, the participants of both groups were informed about the measures that have to be adhered to, i.e., that before the completion they should refrain from alcohol use (for 12 hours), from substantial meals (for 60 minutes) as well as from richly sweet, caffeine, or sour drinks and foods. The participants were also informed that they should not have any dental checkups or interventions in the previous 48 hours, nor clean their teeth in the 45 minutes prior the examination. Saliva was collected between 8.00 and 10.00 a.m.^20^. During the procedure, participants were passively seated in the dental chair with their heads leaning forward. Each participant would be given a polyurethane flask and a glass funnel. They should incessantly allow collecting saliva in their mouths and spitting it into their flasks continuously in the period of 10 minutes. The quantity of saliva was determined by reading the graded flasks after the bubbles disappear to settle saliva. The salivary pH value was measured immediately after collection. Salivary pH values were determined with a pH indicator paper, and the colour change of the indicator would be compared with the existing colour code. The precision of the pH intervals is 0.2 according to the Saliva-Check buffer (GC Corporation, Tokyo, Japan).

### Statistical analysis

Obtained data were processed with standard procedures of descriptive and comparative statistics. The methods of descriptive statistics used in this study were measures of central tendencies, of variability for numeric parameters, and absolute and relative values for attributive markers. Statistical analysis was performed using the IBM SPSS Statistics 20.0. All data were presented as mean±standard deviation (SD). The Shapiro-Wilk test showed normal distribution of the data (p>0.05) for: age, mean pH value, and DMFT index. These numeric variables were analysed using parametric tests (t-test) while number of decayed, missing and filled teeth, and salivary flow rate were tested using Mann-Whitney U-test. The Chi-square test was used to verify possible differences in answers among the groups of participants. The p-values <0.05 were considered statistically significant.

## Results

In the present study, 80 patients were enrolled, 40 in EG and 40 in CG. The obtained results are shown in Table 1. There was no statistically significant difference between EG and CG regarding gender (p=1.000) and age (p=1.000). Prior the beginning of the study, more than 30% of potential participants were excluded because of some reasons.

**Table 1:** Demographic characteristics of the studied population

Parameter	Value	EG	CG
Gender, n(%)	Male	14 (35%)	14 (35%)
p=1.000	Female	26 (65%)	26 (65%)
Age group	Meani±SD	53.00 ± 13.27	53.00 ± 13.27
p=1.000	min - max	23-65	23-65

Regarding the type of disease, 70% of patients were suffering from asthma and 30% were suffering from COPD in the EG. Most of the participants in both groups were allocated into the moderate type (asthma - 53.6%, COPD - 41.7%). As for the length of treatment, most of the participants in both groups had been using inhalation therapy for 5-10 years (asthma - 53.6%, COPD - 91.7%). The question about regularity of undergoing therapy was positively answered by most of participants (asthma - 85.7%, COPD - 75.0%) (data shown in Table 2).

**Table 2:** Descriptive data about the type of disease, length of treatment, and regularity of therapy (%)

Variable	Category	Asthma	COPD	Total
Type of disease	Mild	25.0%	25.0%	25.0%
	Moderate	53.6%	41.7%	50.0%
	Severe	21.4%	33.3%	25.0%
Length of treatment	5-10 yrs.	53.6%	91.7%	65.0%
	11 + yrs.	46.4%	8.3%	35.0%
Therapy	Regular	85.7%	75.0%	82.5%
	when necessary	14.3%	25.0%	17.5%

The EG participants in their treatment of asthma or COPD used a combination of two or more medicines. Frequency of medicine application of the participants in the EG who used inhalation therapy regularly was two times/day. We found out that the EG participants used medicines in the aerosol form (ipratropium bromide + fenoterol 65%) and in the powder form (salmeterol + fluticasone 42.5%, budesonide + formoterol 37.5%, tiotropium 27.5%, beclomethasone dipropionate + formoterol 12.5%, salbutamol 5.0% and formoterol 2.5%). Most of the medicines in the powder form often contain lactose in the form of lactose-hydrate or lactose-monohydrate. Out of 40 participants, 90% used these types of medicaments.

Concerning mouth rinsing we verified that it was: *never* performed by 21.44% of patients with asthma and 50% of patients with COPD; *sometimes* performed by 28.57% of patients with asthma and 0% of patients with COPD; *often* performed by 10.71% of patients with asthma and 8.33% of patients with COPD; and *always* performed by 39.28% of patients with asthma and 41.67% of patients with COPD.

Table 3 shows that there was no statistically significant difference between EG and CG patients in their reports of the type of beverage mostly consumed (p=0.054) or in their use of toothbrush and toothpaste (p = 1.000), dental floss (p=0.274), and interdental toothbrush (p = 0.155). However, statistically significant differences were found in the following responses obtained: interdental toothbrush was reported to have been used by 27.5% participants of CG and no EG participants (p<0.001); fluoride solution for mouthwash was never used by 80% of EG participants compared with 42.5% of CG participants (p<0.001); the lack of information about the proper oral hygiene was reported by more than half of the EG participants - 52.5%, compared with only 12.5% of CG participants (p<0.001); adherence to oral hygiene recommendations was reported by 37.5% of EG participants and as many as 67.5% of CG participants who had been informed about the proper maintenance (p<0.001); scheduling a dental checkup only when there is a problem was reported by 80% of EG participants compared with 17.5% of CG participants (p<0.001).

**Table 3:** Study sample of experimental group (EG) and control group (CG) regarding the dietary history, oral hygiene, fluoride exposure, information about proper oral hygiene, and frequency of checkups at the dentist

	EG	CG	p value
The most consumed beverag			
-water	87.5%	82.5%	p=0.054
-sparkling water, fruit juice, sport beverage	5.0%	17.5%	
-milk, yoghurt	7.5%	0%	
Means for oral hygene			
-toothbrush and toothpaste	100%	100%	p=1.000
-dental floss	15%	27.5%	p=0.274
-interdental toothbrush	0%	27.5%	p<0.001
Oral hygene - toothbrushing			
-more than 2 times a day	20%	27.5%	p=0.155
-2 times a day	55%	62.5%	
-once a day	10%	10%	
-once a week	10%	0%	
-once a month	5%	0%	
Fluoride exposure history - use of fluoride rinse			
-every day	10%	17.5%	p<0.001
-once a week	7.5%	25%	
-once a month	2.5%	15%	
-never	80%	42.5%	
Being informed about the proper maintanance of oral hygene			p<0.001
-yes, and adhere to these recommendations	37.5%	67.5	
-yes, and do not adhere to these recommendations	10%	20%	
-no, not informed about proper oral hygene	52.5%	12.5%	
Dental check-ups frequency			
-every six months	7.5%	45.5%	p<0.001
-once a year	12.5%	32.5%	
-once every 3 years	0%	5%	
	80%	17.5%	

The mean value of the salivary flow rate in the EG was 0.279 mL/min, while in the CG it was higher - 0.496 mL/min. The difference between these values was found to be statistically significant (p<0.001) as well as the one in the mean salivary pH value (p<0.001), which was 6.63 in the EG and 7.23 in the CG (Table 4).

**Table 4:** Mean salivary flow rates (non-stimulated saliva) (mL/min) and mean salivary pH value for both groups

Classification	Group	N	Mean	SD	min	Max	median	p
Salivary flow								
	EG	40	0.279	0.169	0.07	0.71	0.245	<0.001*
	CG	40	0496	0.318	0.06	1.49	0.402	
pH value								
	EG	40	6.63	0.44	5.6	7.6		<0.001**
	CG	40	7.23	0.28	6.8	7.8		

* mann - whitney U test

** t-test

The mean value of DMFT index in the EG was 21.2, while in the CG this value was lower, i.e., 19.4, but no statistically significant difference was found between the groups (p=0.199). However, we found statistically significant differences between groups in the following components: mean number of decayed teeth in the EG was 3.68, while in the CG this value was lower, 1.40 (p<0.001); mean number of missing teeth in the EG was 12.3, while in the CG this number was significantly lower, 6.30 (p<0.001); mean number of filled tooth surfaces in the EG was 5.25, while in the CG this result was higher, 11.6 (p<0.001) (Table 5).

**Table 5:** Decayed, missing, and filled teeth (DMFT) index values and components in experimental (EG) and control groups (CG)

Parameter	Group	N	Average	SD	min	max	p
DMFT	EG	40	21.2	6.89	7	31	0.199**
	CG	40	19.4	5.84	5	32	
Decayed (D)	EG	40	3.68	2.91	0	11	<0.001*
	CG	40	1.40	2.36	0	9	
Missing (M)	EG	40	12.3	6.82	2	25	<0.001*
	CG	40	6.30	4.43	0	24	
Filled (F)	EG	40	5.25	4.75	0	21	<0.001
	CG	40	11.6	5.36	2	23	

* mann - whitney U test

** t-test

Based on the results of DMFT index (p=0.177) and caries lesion (p=0.153) regarding the severity degree of the disease, no statistically significant difference was observed. Also bsed on the results of DMFT index (p=0.541) and caries lesion (p=0.689) concerning the length of the disease, no statistically significant difference was found either.

## Discussion

The present study shows that adults who suffer from asthma and COPD and have been undergoing inhalation therapy for some years have higher caries prevalence, lower salivary secretion rate, and lower pH value compared with healthy individuals.

Although in this study there was a clinically higher value of the mean DMFT index in the EG than in the CG, the difference was not confirmed to be statistically significant (p>0.05), which is in accordance with the findings of another study^13^. However, some researchers^12^ showed that DMFT index in asthmatic group was higher when compared with the control group, resulting from the prolonged use of drugs. In the current study, the mean number of decayed teeth was higher in the EG than in the CG (p<0.001), which was similar to other studies^3^ ^,^ ^15^ ^,^ ^19^ ^,^ ^22^. These results may be the a consequence of either the inhalation medicaments themselves or their application techniques^3^, and could be a determinant factor causing dental caries. In contrast to the aforementioned studies, some authors^5^ did not recognize any connection between the severity of asthma, the period of exposure to medication, and the prevalence of caries. In the presented study, the mean number of missing teeth was higher in the EG than in the CG (p<0.001), which is a finding also confirmed by other researchers^3^. These results support the fact that extraction therapy is the most commonly used therapy for caries in these patients. On the other hand, the mean number of filled teeth in the EG was lower than in the CG (p<0.001). Such distribution of DMFT index structural components may be attributed to the fact that EG participants did not visit their dentists for checkups regularly and did not have adequate dental care, which might be due to the lack of information about proper maintenance of solid dental tissues, the lack of necessary funds, or simply because they were more concerned with primary health issues that may hinder their quality of life and daily activities to a larger extent. Under the circumstances, teeth are permanently covered with non-stimulated saliva. Within the results of this study, the mean value of salivary flow rate in the EG was lower than in the CG (p<0.001). Similar results were received by other authors who considered that the decreased salivary secretion rate in asthmatics is probably caused by the drugs beta-2 agonist and corticosteroids^6^ ^,^ ^23^. Factors that reduce the quantity of saliva can negatively affect oral health, since saliva plays an important role in its preservation^24^. The pH value of non-stimulated saliva in this study was lower in the EG than in the CG (p<0.001), which corroborates the results of other authors^6^ ^,^ ^18^ ^,^ ^23^. Although salivary pH was lower in the experimental group, it was not below the “critical pH” (5.5), which resulted in the enamel demineralisation^25^.

Previous studies have a common statement that patients who suffer from asthma or COPD and who are undergoing inhalation therapy have considerably reduced saliva secretion as well as a lower salivary pH value^6^ ^,^ ^18^, and this could be a determinant factor causing dental caries. However, the question whether these patients have a higher prevalence of caries if compared with healthy controls creates conflicting opinions and contradicted results as well as the question whether the severity and length of the disease correlates with a higher incidence of caries lesions. A group of authors think that there is a higher prevalence of caries in patients undergoing inhalation therapy^1^ ^,^ ^3^ ^,^ ^21^ ^,^ ^22^, which is in accordance with the results of this study. However, another group of authors^16^ ^,^ ^18^ found that patients with asthma have similar dental caries prevalence when compared with the control group, suggesting that this might be due to the fact that this particular group of patients had proper access to dental care^18^. In this research, no statistically significant correlation was observed between caries lesions and the severity of the respiratory disease, since most participants of the EG, i.e., 50%, suffer from moderate forms of diseases (53.56% - asthma and 41.67% - COPD) and a smaller number suffer from mild or severe forms. These relatively small- scale research results should thus be carefully considered, even though they confirm the findings of other studies^3^·^5^·^6^. Some authors believe that a higher prevalence of caries might be linked to the severity of the disease and the consequent high frequency and dosage of the required medicine^19^. In this research we found no significant correlation between caries lesions and the length of the therapy either, which might be linked to the fact that most of the participants in the EG (65%) have been suffering from the disease for 5 to 10 years and may still not have felt the side effects of the therapy. These results are in accordance with the results of other studies^5^. Discrepancies between the mentioned studies regarding the obtained results may be influenced by the number of analysed subjects, diversity of methods, different intensity of the disease as well as the different dozes, types, and efficacy of the medicaments used ^24^.

Previous studies showed increased precipitation of inhaled drugs in mouth cavity^2^, and suggested that it can manifest secondary negative side effects on the oral tissue because of the interaction between the drug metabolism and the saliva^9^. The patients involved in this study used the combination of two or more inhalation medicaments, and at least one of them was in a form of dry powder (DPI). Inhalation medicaments that are in the form of dry powder often contain additional lactose, which can cause a significant decrease in salivary pH values compared with other inhalers that do not contain such sugars. It is suggested that this may be an important cause of enamel demineralization and proliferation of cariogenic bacteria^25^. In the presented study, 90% of EG participants used a type of drug that contained additional lactose. However, some authors^21^ found that the association between lactose in inhalation drugs and caries prevalence was not significant.

Rinsing the mouth with water after medicine application is suggested to be a protective factor in caries development, since it can facilitate cleansing away of residual sugar from tooth surfaces^21^. Although mouth rinsing reduces residual deposition of inhaled anti-asthma drugs, according to some authors, there is no clear evidence that it also reduces dental caries^14^. In the present study, *never rinsing the mouth with water* was reported by 21.44% of asthma patients, and 50% of COPD patients. The majority of individuals in the present study used the inhaler two times/day, at bedtime and morning time. The cariogenic activity increases during the night due to lower salivary flow and lack of masticatory movements^10^.

The interdental toothbrush was used only by 27.5% participants of the CG and none of the EG participants (p<0.001). Moreover, 80% of EG participants never used fluoride solution for mouthwash (p<0.001) compared with a smaller percentage of CG participants (42.5%). It is assumed that lactose in inhaler may lodge between the teeth to cause caries in patients who use inhalation medicines^21^, and the use of interdental toothbrush is therefore vitally important so that the remains of food and sugar could be eliminated. Additionally, rinsing mouth with fluoride-based solutions is also recommended, since fluoride has been shown to significantly reduce the rate of caries^7^.

The data on participants being informed about the proper maintenance and oral hygiene is by no means encouraging, since only 37.5% of the EG participants have been well-informed by their dentists and reportedly respect the preventive measures suggested. However, more than half of the EG participants (52.5%) have not been adequately informed about the proper oral hygiene maintenance. The results show that the participants are likely to have learnt about oral hygiene from their parents or from mass media, and that dental services have not made an effort to provide preventive dental care for this group of patients.

Because of ignorance, anxiousness, or discomfort associated with dental procedures, it is only when there is a problem (pain, most commonly), that 80% of EG participants decide to visit a dentist, whereas this percentage in the CG is considerably smaller - 17.5% (p<0.001). Dental procedures carried out in the EG patients were, in most cases, completed immediately and included tooth extraction. Regular dental examinations in patients undergoing inhalation therapy are of great importance, and they enable a timely diagnostic and therapy of carious lesions, which can eliminate premature tooth extraction and consequent complications (impaired aesthetic appearance, reduced vertical dimension of occlusion, and changes in the temporomandibular joint).

The findings of this study indicate that patients suffering from asthma and COPD who need to undergo inhaled therapy should be more aware of the impact of these drugs on oral health. It is therefore necessary to design and implement preventive dental care for this group of patients as well as to encourage and motivate them in such a way that their quality of life can improve. The results should encourage dentists to educate themselves to discover early changes in the oral tissues in patients on long-term inhalation therapy. Preventive measures should be designed to decrease caries lesions and thus involve. Such measures include advice on dietary habits, saliva stimulation, fluoride supplementation, and appropriate information about oral hygiene maintenance.

Local strategies of preventive dental care, as well as cooperation between dentists and pulmonologists, may considerably improve the oral health of patients undergoing inhaler therapies. Prevention and early diagnosis of these diseases are also very important because of high costs of treatments that might be unnecessary if adequate preventive measures were taken. The improvement can be achieved if programmes of dental health care and prevention are implemented on a larger scale - within the whole country or region.

## Conclusion

We found that patients undergoing inhalation therapy face increasing risk of dental caries due to a lower salivary flow rate and pH value associated with the inhalation therapy. In order to stop the disease progression in the hard dental tissue of the mouth cavity, it is necessary to apply adequate preventive measures and positively motivate their continuous application in this caries-risk population.

A better mutual collaboration among dentists, general practitioners, and pulmonary specialists would contribute to improve general oral and dental health in these patients.
